# Supplementary material for: Spiking neural state machine for gait frequency entrainment in a flexible modular robot
Source: PLoS One. 2020 Oct 21;15(10):e0240267. doi: 10.1371/journal.pone.0240267 (PMC7577446; doi:10.1371/journal.pone.0240267)
Supplement: Attachment — Submitted filename: responses.pdf [file pone.0240267.s005.pdf]

Reviewer	Place	Comment	Response
Editor		The manuscript requires revision to provide details of the model and its justification, and should be put into the context of the contemporary literature. The introduction makes it seem that computation with spiking neural networks is a novel concept (by contrasting it to ANNs in the second paragraph of the introduction but not mentioning prior literature) developed here. - This needs major rephrasing	The introduction has been rewritten and substantially clarified, and additional information necessary for replication has been added, including a new SI file, a Julia notebook containing the full simulation source code.
Reviewer 1		The paper describes that robot models legged locomotion, that is much more complex behavior than just flexion/extension of legs. It looks like model of worm locomotion for me.	The robot's locomotion is tripod-stable, which is rare in biological quadrupeds. We did not intend to give the impression that the gait is a model of quadruped locomotion; some wording has been revised with this in mind.
Reviewer 1	Lines 17, 52, …	The paper would be stronger if the authors could avoid using electrical engineer jargon. Examples: ‘converted ANNs’; ‘SR latch’.	We have revised the text to eliminate or explain such jargon, in particular replacing the unnecessary term "flip-flop" with the (in this context) synonomous "latch", which we had already introduced
Reviewer 1	Intro	For the sake of completeness, the discussion of the bistability in neuron models should include also the study by [...]	Thank you for the reference recommendations. They present a compelling picture of the dynamical bifurcations underlying bistability in single neurons. This work is somewhat peripheral to ours in that we are mainly concerned with constructing a bistable circuit from neurons which are monostable, but it is worth mentioning single-neuron bistability in the context of dynamical properties which may be used for computation outside the scope of this paper at the end of section 2.1.
Reviewer 2	Sec 1.	There is a large body of literature describing models of locomotor CPGs using populations of spiking neurons, such as the work of Eve Marder and Ilya Rybak and others. These warrant discussion. As the paper currently reads, a naive reader could think that the use of spiking neurons for CPG models is a novelty of the paper.	The new introduction attempts to be clearer about this point.
Reviewer 2	Sec 1.	Further, there are several examples of spiking neural networks used for the control of robots (a simple google scholar search will reveal several).	The rewritten introduction is clearer about this fact, with existing citations made more prominent.

Reviewer	Place	Comment	Response
Reviewer 2	Sec 1.	The biggest issue of the paper is, that the introduction makes it seem that computation with spiking neural networks is a novel concept (by contrasting it to ANNs in the second paragraph of the introduction but not mentioning prior literature) developed here. - This needs major rephrasing.	The introduction has been rewritten with this in mind.
Reviewer 2	Sec 2.	The mechanisms of rhythmogenesis in the presented controller is seemingly based on temporal summation of synaptic inputs and the individual modular building blocks are not intrinsically rhythmogenic. While this is a possible mechanism for the generation of rhythmicity, there are many others and it is not the most likely one. In mammals, for example, the view is that each limb-specific circuit is capable to produce a rhythm itself (since a rhythm can be elicited in the hemicord) and slow persistent ion channels likely underly rhythmgenesis. The chosen mechanism has to be discussed in connection with the literature.	We have added a paragraph to section 2 clarifying the mechanism of rhythmogenesis in our system, in particular the fact that it is not biologically typical, just plausible and readily compatible with the engineering approach which we are applying.
Reviewer 2	Sec 2.	The article refers to the individual modules as oscillator modules or CPG modules and even CPG networks, which is clearly misleading.	We have made the terminology more consistent throughout, removing uses of the word "network" to describe the module and avoiding the confusion of having used the phrase "oscillator module" because it *is* an oscillator vs. "CPG module" because it is used to *construct* a CPG.
Reviewer 2		The two types of oscillations described in the paper, tonic neural firing vs. neural bursting, should be clearly distinguished in the text.	A note to this effect has been added where the bursting-like activity is introduced.
Reviewer 2	Figure 2, 7	Figures showing neural activities should include the synaptic currents to illustrate the mechanisms of rhythmogensis.	Figure 2 has been replaced with a new figure and a more thorough description of the mechanism of rhythmogenesis.
Reviewer 2	Sec 3.	It is not clear why the robustness and sensitivity of only the three-cell building block is analyzed. The robustness of the final network would be of much greater interest, especially since the single module seems to lack the ability to generate bursts.	The idea is that the robustness of the three-cell building block is inherited by the larger network due to the modular system design. This is now explicitly mentioned in the text.
Reviewer 2	Sec 3.	It is not exactly clear how function of the latch-circuit was operationalized for the sensitivity/discriminant analysis. Was only the presence of a limit-cycle (> 5ms) used= What about theability to change states using external inputs?	Only the existence of the limit cycle is relevant because it is not possible to eliminate the resting state without adding a bias current. That is, there is no way to create a module that can't change states in response to inputs as long as the limit cycle exists. Section 3.1 is now more explicit about this.

Reviewer	Place	Comment	Response
Reviewer 2	Sec 3.	Results are mostly described qualitatively and lack quantitative detail (e.g., what are the axes in Fig 4).	The axes of figure 4 represent unitless *relative* deviation (hence the percentages) along the axes uncovered by linear discriminant analysis. This is analogous to PCA, but using already-known labels. The text has been edited to explain this more clearly.
Reviewer 2	Sec 4.	The results are unlikely to be reproducible. For example, I couldn't find the parameters for the synaptic conductances between the modules, parameters used in Fig 3 are not given etc. All parameters should be clearly specified and easy to relate to the described results. Furthermore, the source code for all simulations (including all figures) should be published with the paper (e.g. GitHub, ModelDB).	A new table of parameter values has been added, and the simulation code has been gathered into a Jupyter notebook, which is attached as a new SI file.
Reviewer 2	Intro	Neuromorphic computation is introduced as using spikes. In the next paragraph neuromorphic machine learning is equated to artificial neural networks. This is confusing.	This error is avoided in the rewritten introduction.
Reviewer 2	Line 123	"model parameters have biophysical meaning" - This is a bit of a stretch, esp. when compared to Hodgkin-Huxley type models.	This has been replaced with "model parameters are physically interpretable". The intention was to contrast with e.g. FitzHugh-Nagumo neurons, whose parameters don't correspond to any physical quantity.
Reviewer 2	Lines 169 - 172	The selected model parameterizations taken from Izhikevich should be explained in more detail. What is their spiking patterns. Why were they selected.	Several sentences have been added here explaining that the choice of parameter values is somewhat arbitrary because the method extends flexibly to other neuron models with qualitatively similar dynamics. We chose these types because they do not produce tonic spiking or bistability, so we can implement these functions in circuitry instead for additional robustness.
Reviewer 2	Line 203	This is an odd analogy. Especially since that would relate to the original non-leaky integrate and fire neuron. While the neuron at hand is the extension of the leaky version.	The neural circuit conceptual model has been explained in more detail. In particular, channels other than at synapses are now explicitly mentioned, and the relationship to Hodgkin-Huxley conductance-channel models has been explained.
Reviewer 2	Fig 3.	It is not clear to me what the authors try to illustrate. At the very least the exact parameters used should be indicated.	Figure 3 was intended to illustrate the fact that parameter randomization does not affect the qualitative behavior of the module - in fact, we don't know the parameters that were used. We have attempted to explain this better by adding more text and presenting the figure slightly later so that it can refer to the parameter randomization more explicitly.
